# An evaluation of the knowledge and perceptions of pharmacy students on pharmacovigilance activities in Nigeria

**DOI:** 10.1186/s13104-017-2586-9

**Published:** 2017-07-12

**Authors:** Kanayo P. Osemene, Margaret. O. Afolabi

**Affiliations:** Department of Clinical Pharmacy and Pharmacy Administration, Faculty of Pharmacy, Obafemi Awolowo University, Ile-Ife, Osun Nigeria

**Keywords:** Pharmacy students, ADRs, Pharmacovigilance, Activities, Nigeria

## Abstract

**Background:**

The use of the modified-prescription event monitoring technique has facilitated the understanding and reporting of pharmacovigilance (PV). However, in Nigeria, PV activities are largely misunderstood. Furthermore, there is a dearth of information on the knowledge and perceptions of pharmacy students on PV activities in relation to demographics. This study investigated and assessed the knowledge and perceptions of pharmacy students about pharmacovigilance as well as the demographic factors that are related to pharmacovigilance activities.

**Methods:**

A cross-sectional survey was conducted among final year pharmacy students in three universities in months of January and February, 2016 with permission from the institutions and with written consents from 342 respondents. Pre-tested questionnaire was used to elicit information on the study objectives. Data were analysed using appropriate descriptive and inferential statistical techniques.

**Results:**

The study revealed that the mean score on knowledge of pharmacy students on pharmacovigilance activities was 4.3 ± 0.18 which was significant according to gender (P < 0.001), students’ university (P < 0.001), and previous exposure to PV subjects (P < 0.001). Sixty-four percent of the students had positive perceptions about PV activities which was significant at P < 0.00 according to gender and their various universities. Less than half of the respondents 165 (48.2%) were able to correctly name the organisation that collates and document ADR reports in Nigeria. Only 21 (6.1%) of the respondents gave the correct answer to whether or not all possible ADRs of a drug can be determined during clinical trials or during pre-marketing phase of drug assessment. About 204 (59.7%) of the respondents erroneously believed that adverse reactions caused by cosmetics should not be reported.

**Conclusions:**

Respondents have inadequate knowledge of PV activities. Therefore, pharmacy student educators should enhance students’ knowledge about PV through training, during clerkship, and lay more emphasis on relevant PV courses in the Pharmacy Curriculum.

**Electronic supplementary material:**

The online version of this article (doi:10.1186/s13104-017-2586-9) contains supplementary material, which is available to authorized users.

## Background

Post marketing surveillance of drugs is important because it is impossible to determine all adverse effects of drugs before they are put to use [1, 2]. Hence knowledge in pharmacovigilance (PV) activities and adverse drug reactions (ADRs) reporting become necessary in this regard. Such knowledge should be taught in the university at the undergraduate level while more knowledge should be acquired by health care professionals during practice [3]. However, the level of knowledge of undergraduate students in health-related discipline such as pharmacy, about ADR reporting and PV activities has not been fully studied in Nigeria. Since training and quality of knowledge are important for good professional practice, would-be health professionals such as student doctors, pharmacists among others are expected to be knowledgeable enough in their chosen career in order to render quality professional service that could guarantee favourable therapeutic outcomes by improving patients’ quality of life.

Arguably, health workers, especially pharmacists, are believed to be better placed to report ADRs because they are easily accessible, operate flexible and long working hours and easily come in contact with a lot of patients [4]. In addition, pharmacists are expected to play a leading role in the monitoring and reporting of ADRs. In this regard, the role of pharmacist in Nigeria about PV activities are to: monitor drug use, misuse, and abuse at all times in order to ensure proper medication use [5]. They are to direct ADR reports to the National Agency for Food Drug Administration and Control (the official organization that collates ADRs reports in Nigeria), conduct post-marketing surveillance, report medication errors and suspected counterfeit or substandard drugs; and monitor drug utilization [6]. However, the knowledge and perception of community pharmacists in Nigeria towards ADR reporting have poor [5]. This poor knowledge among community pharmacists in Nigeria may be due to lack of appropriate training in the undergraduate curriculum [5].

Meanwhile, it was documented that pharmacists’ involvement in the reporting of ADRs improved the volume and quality of reports in developed countries [7]. This appears not to be so in Nigeria [5].

Nevertheless, outcomes from similar studies conducted in developed countries revealed among other things, that pharmacy students had inadequate knowledge, poor awareness but positive attitude of PV activities [8–19]. However, only one of such studies showed that there was significant difference in perceptions of the students on ADR reporting [20]. In that study, pharmacy students’ inadequate knowledge on PV activities was attributed to inadequate PV courses in the Faculty of pharmacy curricula [21]. Furthermore, two independent studies assessed the knowledge and attitude of would-be healthcare professionals towards PV and ADR reporting and found that their knowledge of PV activities were low and average respectively [8, 20]. In all, none of the above-mentioned studies, evaluated the students’ PV knowledge across institutions. To the best of our knowledge, no known study in Nigeria assessed the relationship between the demographic characteristics of would-be healthcare professionals and knowledge of PV.

This study was therefore aimed at investigating the knowledge and perceptions of pharmacy students about pharmacovigilance with a view to providing information on their knowledge and perceptions about PV as well as the demographic factors that are related to PV activities. The study will contribute to the existing literature in light of the scarcity of relevant data on pharmacy students’ knowledge about PV activities in Nigeria.

## Pharmacy education in Nigeria

Pharmacy was first established as a Department in the Nigerian College of Arts, Science and Technology, in 1957 and pharmacy curricula was dominated by compounding and dispensing. Later, courses like pharmacology, toxicology, and pharmaceutical microbiology were added to the curricula [22]. In 1962, the University of Ife (now Obafemi Awolowo University) was established and it took over the training in pharmacy in a Department in the Faculty of Science. It continued to award Diploma in Pharmacy till 1965 while concurrently running a programme for the Bachelor of Pharmacy which was started in 1963. As at now, candidates applying into the first year of the Bachelor of Pharmacy (B. Pharm) programme are required to have credit passes in five (5) subjects including english, mathematics, physics, chemistry and biology in the Senior Secondary Certificate Examination or its equivalent [23].

The B. Pharm. Degree is a 5 year program which was designed in such a way that the basic sciences are taught in parts 1 and 11; while in parts 111, IV and V, students are taking through clinical or professional training. Clerkship and externship programmes are conducted in their final year (part V), where they are expected to register for clinical pharmacy courses such as pharmacokinetics, therapeutics, clinical pharmacy clerkship, chemotherapy, drug information among others [24]. There is no stand-alone course in medication safety. In clinical pharmacy clerkship, for instance, the students are posted to hospitals to conduct ward rounds with other health care workers, review prescriptions (where necessary), monitor patients’ drug use, learn vital issues on medication safety and document therapeutic outcomes. Also, pharmacy students are exposed to pharmacy-supervised experiences (externship) in government approved community pharmacies as partial requirement for the award of the Bachelor of Pharmacy (B.Pharm) degree. The main goal of the entire exercise is to among other things, train the students in practical clinical pharmacy activities such as scrutinizing prescriptions for completeness, monitor drug–drug interactions, document actual and potential drug-related problems [25]. The mode of teaching is didactic in nature.

The B. Pharm degree is unclassified with a pass mark of 50% in all courses except in Dispensing, and Forensic Pharmacy and Pharmacy Ethics, where the pass mark is 60%. Upon successful completion of the part V Bachelor of Pharmacy examinations, students are inducted into the pharmacy profession and are required to proceed for a 1-year compulsory internship program in approved or designated centres/institutions [25, 26]. The prospect of getting a job after graduation as a pharmacist is very high in Nigeria.

## Methods

### Setting

The study was carried out in three universities (out of five) in southwestern Nigeria that offer pharmacy as a course and their curricula are unified [27].

### Design and study population

The study was a cross-sectional survey and was undertaken with due permission from the institutions and with written consents from respondents. The list of the respondents which was obtained from their Deans served as the sample frame. Due to time and cost, the three universities were purposively selected in spite of the fact that they are in the same geographical zone and close to the base of the researchers.

### Inclusion criteria

Only final year (Part V) pharmacy undergraduate students who would have taken requisite courses in medication safety and clinical pharmacy took part in the study.

### Exclusion criteria

Pharmacy students in lower classes (Parts 1, 11, 111 and IV) and those in the postgraduate (PG) level were excluded from the study because the former had no clinical exposure and have not taken courses in PV subjects. For the later, not all the PG students had their first degrees in Pharmacy or hold Pharm.D degrees. Some of them read allied pharmacy courses such as botany, zoology, chemistry, and microbiology in their first degrees and decided to pick higher degrees in pharmacology, pharmaceutical chemistry, pharmaceutical microbiology, phytochemistry among others. These categories of PG students are not regarded as core pharmacy students.

### Questionnaire design and questionnaire administration

The study adapted the survey instruments used in similar studies to measure knowledge; attitude and perception about PV activities elsewhere [28–32]. However, some of the survey instruments (questionnaire, Additional file 1) were modified to suit the Nigerian environment. A total of 31-item survey instrument of three domains, namely demographics, knowledge and perceptions of final year pharmacy students on ADRs reporting and pharmacovigilance were designed.

The first part contained 6-item survey questions on demographics such as age, current university, gender, and questions on whether the students have ever heard of the terms adverse drug reaction, pharmacovigilance and if they have taken any course that was related to pharmacovigilance.

The second part contained questions on core issues which were designed to measure the students’ basic knowledge about pharmacovigilance and adverse drug reaction reporting. The questions had their answers in multiple choice format and students were asked to choose the correct answers to the questions. Knowledge on ADR reporting was measured using 10 true or false items. A weighted score of 1 was assigned to each correct answer and 0 for each wrong answer. The maximum score obtainable for the 10-item survey was 10 and the minimum score was 0. Each student’s knowledge about PV activities was assessed by adding all correct answers over the maximum obtainable score. Results obtained were in percentages. Since the pass mark for pharmacy courses in Nigerian universities is 50%, any respondent who got less than 5 questions correctly out of 10 the questions in the survey item (<5/10) was deemed to have performed poorly and therefore considered to have poor knowledge about PV activities. Meanwhile, undergraduate pharmacy students are graded in Nigeria as follows: scores <50% (poor), 50–60% (good), 60–69% (very good) and >70% (excellent).

The third section of the questionnaire contained 15 survey items which were designed to evaluate the perceptions of the pharmacy students toward adverse drug reporting. Students’ perceptions of ADR reporting were measured on a 5-point Likert scale. This showed their level of agreement or otherwise on statements about ADR monitoring and reporting and pharmacovigilance in Nigeria as 1 = strongly disagree, 2 = disagree, 3 = slightly agree, 4 = agree, and 5 = strongly agree. The questionnaires were structured and contained mostly closed-ended questions whose answers were not mutually exclusive.

In filling the questionnaire, the students were asked to work independently by avoiding any interaction amongst them and to refrain from consulting reference materials. This was done to ensure that actual individual students’ knowledge about PV activities was employed in filling the questionnaire. The questionnaire was hitherto pre-tested among 10 final year pharmacy students who did not take part in the study and the comments of the pharmacy students were used to make necessary corrections and modifications such as the reframing of some questions into multiple choice format and rewriting of some abbreviations such as ADRs and PV in full for clarity. The pilot testing of questionnaires was to ensure that the concepts being measured were understood and that the answers provided were germane to the concepts (face validity). Effort was also made to ascertain if the respondents to the piloted test understood what was asked for as contained in the questions and if the questions actually covered what was examined on PV activities (content validity). The Cronbach alpha test for each set of questionnaire was determined and the final Cronbach alpha value for the entire questionnaire was computed. The questionnaires were administered to the students by the researchers by face to face meeting in the presence of their course coordinators and about 15 min later, completed questionnaire were collected by the researchers. The questionnaire for the study is presented as additional file 1.

### Statistical analysis

Data on demographics, knowledge and perceptions of pharmacy students’ toward ADRs about PV activities were analyzed with IBM SPSS Statistics 20 and presented in percentages, means, standard deviations and median at 50% percentile.

Knowledge score of the respondents about PV was computed and presented in percentages and means. The association between knowledge score and the demographic variables of gender and the presence of PV courses in the universities was determined using the independent Student t test. While the association between respondents’ knowledge of PV with their ages and respective universities were examined using the analysis of variance (ANOVA).

Perceptions of the final year pharmacy students about PV activities were presented with descriptive statistics such as frequency and median at 50% percentile. These were used in describing their opinion to specified statements in ordinal scale. The ranked variables were evaluated using Kruskal–Wallis and Mann–Whitney U tests as appropriate at P < 0.05.

## Results

A total of 342 final year pharmacy students properly filled the questionnaire. Data obtained were used for analysis. Response rate was 98.3%. The Cronbach alpha values for the first, second and third set of questionnaire were 0.73, 0.72, and 0.71 respectively. Test of reliability of the final copy of the questionnaire gave a Cronbach alpha value of 0.72. Majority of the final year pharmacy students were males (65.2%). Most respondents were between 21 and 30 years of age with a mean age of 25.5 ± 4.3 years. Only 2.6% of them were above 30 years of age. The mean knowledge score in pharmacovigilance and adverse drug reporting by the final year students was 4.25 ± 0.18. There were significant differences in the mean scores in the knowledge domain by gender (P < 0.001) as well as the current universities attended (P < 0.001). Also, there was a significant difference in the mean scores of pharmacovigilance knowledge between the respondents who had taken some courses in pharmacovigilance and those who did not (P < 0.001). Furthermore, there was a significant association between those respondents who had taken courses related to pharmacovigilance, with their current universities (P < 0.001).

The relationship between the mean knowledge score of Nigerian final year pharmacy students with their demographic characteristics is presented in Table 1.

**Table 1:** Relationship between the knowledge score of Nigerian final-year pharmacy students with their demographic characteristics (N = 342)

Demographics	Response rate, n (%)	Mean knowledge score (SD)	Values	*P*
Gender				
Male	223 (65.2)	4.1 (1.20)		
Female	119 (34.8)	4.4 (0.98)	10.65	0.01^a^
Age (years)				
21–24	120 (35.1)	4.8 (0.70)		
25–30	213 (62.3)	6.2 (0.84)		
>30	9 (2.6)	4.6 (0.72)	129.330	0.01^b^
Pharmacovigilance course				
Yes	263 (76.9)	7.3 (0.22)		
No	79 (23.1)	4.7 (0.13)	16.637	0.01^a^
No. of students in each university				
1 (n = 110)	107 (31.3)	5.1 (1.03)		
2 (n = 143)	141 (41.2)	6.4 (0.99)		
3 (n = 94)	94 (27.5)	4.3 (1.01)	142.041	0.012^b^

^a^Independent t test

^b^Analysis of variance

In Table 2, less than half of the respondents 165 (48.2%) were able to indicate the organisation (NAFDAC) whose prime duty is to collate and document various ADRs reports in Nigeria. Also, respondents’ knowledge on what the terms adverse drug reactions and pharmacovigilance mean, were 169 (49.1%) and 173 (50.6%) respectively. Very poor performance by respondents in the knowledge domain was recorded from the answer to question on the type of ADRs to be documented 118 (34.5%). Also, only half 174 (50.9%) of the pharmacy students knew that all cases of ADRs should be reported. However, only few respondents 21 (6.1%) could provide correct answer to whether or not, all possible adverse reactions to any drug could be known in the pre-marketing phase or during clinical trials.

**Table 2:** Final-year pharmacy students’ knowledge concerning ADRs reporting in Nigeria (N = 342)

Survey items	Respondents who got the correct
	Answers to survey questions (items)
	No. (%)
1. What do you understand by the term adverse drug reactions?	168 (49.1)
2. What is pharmacovigilance?	173 (50.6)
3. Which types of ADR should be documented?	118 (34.5)
4. What type of ADR reporting system do we have in Nigeria?	143 (41.8)
5. All ADRs should be reported	174 (50.9)
6. There are no guidelines for reporting ADRs in Nigeria	167 (48.8)
7. What types of ADRs do you know?	147 (43.0)
8. ADRs caused by herbal medicines are neither documented nor reported	176 (51.5)
9. All ADRs are known before a drug is marketed	21 (6.1)
10. Which organization should case of ADRs be reported to in Nigeria?	165 (48.2)

Almost all the students 339 (99.1%) indicated that they have acquired enough knowledge to enable them report ADRs (Survey Statement 1 in Table 3). Also, most of the students 318 (92.98%) believed that incorporating more clinical courses into the pharmacy curriculum will improve their knowledge to report ADRs (Survey Statement 12). A very high number of the students 321 (93.86%) were of the opinion that pharmacists’ involvement in reporting ADRs would impact positively on pharmacovigilance activities (Survey Statement 11). About two-third of the students 211 (61%) believed that late or non-reporting of ADRs could pose major health problems (Survey Statement 4). Just a little above half of the respondents 190 (55%) believed that ADR reporting is an integral part of pharmaceutical care (Survey Statement 9). A reasonable high number of the students 251 (73.4%) were of the opinion that monetary incentives to health workers may improve ADR reporting (Survey Statement 6). More than half 204 (59.7%) of the respondents wrongly believed that adverse reactions caused by cosmetics should not be reported (Survey Statement 15). A majority of the students were of the opinion that if practical demonstration of how to report ADRs was illustrated in class by lecturers, reporting rate could increase when they become graduates from their respective universities (Survey Statement 13). More than half 189 (55.26%) of the students claimed that they can perform ADR reporting during clerkship programmes (Survey Statement 7).

**Table 3:** Final-year pharmacy students’ perceptions about ADR reporting in Nigeria (N = 342)

S/no	Survey statements	SD 1	D 2	SLa 3	A 4	SA 5	Median (50-per)	Mean	Kruskal–Wallis	Mann–Whitney U	P value
1	I believe that I have acquired enough knowledge to enable me report ADRs	1	1	1	126	213	5.00	4.6	262	5593	0.01
2	Pharmacovigilance should be taught at all levels in pharmacy schools	120	60	50	72	40	2.00	2.6	289	150	0.02
3	Majority of ADR reports should come from pharmacists	108	91	80	32	31	2.00	2.4	300	672	0.02
4	I believe that non- reporting of ADRs could pose major health problems	61	50	20	88	123	4.00	3.5	289	238	0.01
5	I believe that ADRs caused by herbal medicines should be reported	130	100	75	7	30	2.00	2.14	284	326	0.04
6	Monetary incentives to health workers may improve ADRs reporting	16	65	10	101	150	4.00	3.9	260	1845	0.02
7	Pharmacy students can perform ADR reporting during their clerkship	35	43	75	80	109	4.00	3.54	283	350	0.03
8	I believe that government could improve ADR reporting	94	64	80	95	9	3.00	2.59	299	488	0.02
9	I believe ADR reporting is an integral part of pharmaceutical care	31	68	53	69	121	4.00	3.53	286	119	0.01
10	Pharmacists should not be barred from reporting ADRs	154	121	36	11	20	2.00	1.89	248	1794	0.01
11	I believe if pharmacists report ADR PV activities will improve	5	2	14	198	123	4.00	4.26	234	238	0.04
12	Disclosure of identities of ADR reporters would increase reporting	10	5	9	53	265	5.00	4.63	215	8687	0.01
13	I believe that if the identities of ADR reporters are not disclosed reporting rate will decrease	58	21	44	98	121	4.00	3.59	288	119	0.02
14	Pharmacovigilance should be taught only at higher levels in pharmacy schools	32	17	20	88	185	5.00	4.04	245	3927	0.01
15	I believe that adverse reactions caused by cosmetics should be reported	126	78	29	16	93	2.00	2.63	300	95	0.03

Likert scale key: strongly disagree (1), disagree (2), slightly agree (3), agree (4) and strong agree (5)

P < 0.05 was considered significant

The computed Kruskal–Wallis and Mann–Whitney U values for all the survey statements in Table 3 were significant at P < 0.05.

## Discussion

The knowledge of Nigerian pharmacy students on the reporting of ADRs and pharmacovigilance activities is inadequate. This result agrees with the findings of previous studies conducted elsewhere [8–13, 20]. Low knowledge of PV could trigger the need to review curriculum in terms of expanding or increasing the course content of PV courses vis-a-vis clinical courses relating to PV. The significant differences in the mean knowledge score on PV activities by gender (P < 0.001) could be attributed to the differences in their level of interest in PV subjects (11). The study revealed that female students had better knowledge of ADR reporting than their male counterparts. This finding is at variance with the result of a similar study [33]. Furthermore, most pharmacy students’ inability to correctly answer Survey Statement 9 in Table 2 (all ADRs are known before a drug is marketed) is a flaw in their knowledge of PV activities, in spite that they claimed to have acquired enough knowledge to indeed monitor and report ADRs (Survey Statement 1 in Table 3). The misconceptions on whether it is possible to detect all side effects caused by drugs in the pre-marketing phase of clinical trial could have been addressed by pharmacy educators or instructors. However, reasons for pharmacy students’ poor knowledge of ADR monitoring could be due to absence of enough PV courses in their universities’ curricula [10], and lack of adequate hands-on training during clerkship [34] on how ADRs reporting forms should be used. Variations in knowledge of PV activities amongst students in the three universities which was significant at P < 0.001 constitute notable findings of this study. These variations could be attributed to differences in the content of PV-related courses of each university in spite of the fact that the curricula of the faculties of pharmacy are unified [27]. Bridging this knowledge gap could be achieved by deliberate increase in the course content in PV subjects which could be achieved during curriculum review. Curriculum review is done whenever it is necessary. To review the curriculum, a committee is set up by the Faculty Board of Examiners (FBE) which is made of members from various departments in the faculty. The outcome of the review exercise is deliberated upon by FBE and recommended to the Business Committee of Senate (BCS) for further processing. The BCS vets it and passes it on to Senate for final approval.

Nevertheless, most of the students disagreed that adverse reactions caused by cosmetics should be reported (Survey Statement 15). Cosmetics, arguably represent the commonest and most widely used product all over the world. Most times they are regarded as daily needs. However, more often than not, a lot of people do not really have a clear understanding of what cosmetics are. The main issue is whether a product is a cosmetic or a drug or both. Sometimes, a product can be a cosmetic and a drug. This happens when the product has two or more intended uses. For instance, antidandruff shampoo is used for dual purposes. The shampoo cleanses the hair as a cosmetic agent while antidandruff is used to treat dandruff and therefore is a drug. Among other cosmetics/drug combinations are toothpastes that contain fluoride, deodorants that are also antiperspirants, and moisturizers and makeup marketed with sun-protection claims [35]. Anyway, this notable misconception associated with ADRs reporting arising from the use of cosmetics could be as a result of the students’ lack of experience in ADR reporting guidelines. Hence, they did not know what and what not to be reported.

Most of the students were of the opinion that monetary incentives to health workers could improve ADR reporting. This result is similar to the findings of Inman [36] who reported that lack of monetary incentives to ADRs reporters was one of the prominent “seven deadly sins’’ that caused ADR under-reporting. Monetary incentives for reporting ADRs have influenced the attitudes of some health workers toward ADRs reporting [36]. However, commercializing such a mandatory and fundamental health activity could be prone to abuse and froth with immense danger because the quest to make money could override the desire to render quality service. Therefore, other strategic approaches to incentivize pharmacists to report ADRs include but not limited to giving healthcare promoters access to ADRs data, making ADR reporting forms and data easy to obtain, interpret, and use. Also, improvement in infrastructural facilities such as internet connectivity, epileptic power supply could help especially in developing countries [37]. In addition, other non-monetary incentives could come in form of flexible work hours, training, the creation of a pleasant work environment, and granting sabbaticals to ADR reporters [38].

A remarkably high number of the students believed that pharmacist participation in ADRs reporting would have positive impact on PV activities. This finding is comparable with the result obtained by various researchers [34, 39]. In literature, it was revealed that ADR monitoring and reports emanated from community and hospital pharmacists [39–41]. Pharmacists in these settings come in contact with patients easily who either come to fill prescriptions or for consultations. In the course of this pharmacist-patient interactions, pharmacist could evaluate the patients’ medication profile and give professional advice that could ensure better medication outcome even at reduced cost.

Pharmacy students who responded to survey item 9 in the perception domain rightly noted that PV activities could be regarded as an integral part of pharmaceutical care. Pharmaceutical care which was an American concept has added a new dimension to the teaching and to the practice of pharmacy profession world-wide [25]. In addition, a comprehensive knowledge in clinical pharmacy courses and practical exposure to cases in the hospitals which the clerkship programme is meant to offer remains a unique opportunity that pharmacy students must utilize in order to understand and acquire the skills required for quality ADR reporting. The clinical exposure of the students would enhance their willingness to report ADRs because they would be familiar with PV cases in the hospitals and learn from their tutors how to deal with such cases in terms of reporting it to the appropriate PV centres.

Nevertheless, this study provided information on the knowledge and perception of pharmacy students about PV and the demographic factors that are related to PV activities in Nigeria. The study also added to the body of knowledge by proffering ways of enhancing the understanding of PV activities by university students. The major strength of this survey is that international readers could use the results as a basis for comparison with similar studies carried out abroad. In addition, the study focused on an issue that has not been adequately studied in Nigeria.

## Limitations of the study

However, some prominent limitations of the study were that the universities examined were too few compared to the number of universities in Nigeria. The purposive selection of the three universities would have created some element of bias. A random sampling could have reduced such bias. The study did not include post graduate pharmacy students; and limitations associated with questionnaire-based studies such as subjective responses cannot be completely ruled out. Hence, this study may not generalizable. In spite of all this, results obtained from this study would provide information for further studies in Pharmacovigilance as well as provide clear intervention targets for pharmacy student educators.

## Conclusion

The study revealed that Nigerian pharmacy students have inadequate knowledge of pharmacovigilance activities; but have positive perceptions towards ADR reporting. The mean score on students’ knowledge of PV activities was significant according to gender, students’ university and previous exposure to PV subjects. Periodic curriculum review with the motive of increasing the content of PV courses could enhance the students’ knowledge about PV. Also emphases should be laid on hands-on training and more focus should be paid to the clerkship and externship programmes because they afford the students the opportunity to have real life experiences with patients.

## Suggestions for further research

Further studies could focus on the evaluation of curricula to determine areas of differences that must be addressed in order to enhance pharmacy students’ knowledge about PV. In order to generalize the results of this study, it is strongly suggested that studies with larger samples and including students from more universities should be undertaken in the future. In addition, PV knowledge of post-graduate students who have the first degrees in pharmacy could be assessed too.

## Additional file

**Additional file 1.** Questionnaire.

## Abbreviations

PV: pharmacovigilance
ADRs: adverse drug reactions
NAFDAC: National Agency for Food Drug Administration and Control
B. Pharm: Bachelor in Pharmacy

## References

[1] Kharka M, Bowalekar S (2012). Knowledge, attitude and perceptions/practices (KAP) of medical practitioners in India towards adverse drug reaction (ADR) reporting. Perspect Clin Res..

[2] Stricker BH, Psaty BM (2004). Detection, verification, and quantification of adverse drug reaction. Br Med J.

[3] Pankaj G, Aaditya U (2011). Adverse drug reaction reporting and pharmacovigilance: knowledge, attitude and perceptions amongst resident doctors. J Pharm Sci Res.

[4] Trinca CE (1995). The pharmacists progress toward implementing pharmaceutical care. Am Pharm.

[5] Osemene KP, Ayeni MI, Afolabi MO (2012). The role of community pharmacists in monitoring adverse drug reactions in Nigeria. J Health Ser Res..

[6] Owolabi T. Role of NAFDAC in drug regulation. Medicine regulation claims. From concept to launch. In: Proceedings of an international conference organized by centre for drug discovery, development and production (CDDDP). Faculty of Pharmacy, University of Ibadan in conjunction with Reckitt Benckiser, UK. p. 35–42.

[7] Knapp KK, Katzman H, Hambright JS, Albrant DH (1998). Community pharmacist intervention in a pharmacy benefit contract. Am J Health Syst Pharm.

[8] Sivadasan S, Sellappan M (2015). A Study on the awareness and attitude towards pharmacovigilance and adverse drug reaction reporting among nursing students in a private University in Malaysia. Int J Curr Pharm Res..

[9] Etminani M, Isfahan SM, Amin R, Mehrdad A, Leila K, Kaveh E (2013). Adverse drug reactions: knowledge, attitude and perceptions of pharmacy students. J Pharm Care.

[10] Showande JS, Fakeye TO (2013). The Concept of adverse drug reporting: awareness among pharmacy students in a Nigerian University. Int J Med Update.

[11] Elkalmi RM, Hassali MA, Ibrahim MI, Widodo RT, Efan QM, Hadi MA (2011). Pharmacy students’ knowledge and perceptions about pharmacovigilance in Malaysian Public Universities. Am J Pharm Edu.

[12] Kalari S, Dormerumo M, Zyenigorodsky O, Mohan A (2011). Pharmacy Student Perceptions of Adverse Event Reporting. Am J Pharm Edu..

[13] Gavaza P, Bui B (2012). Pharmacy students’ attitudes toward reporting serious adverse drug events. Am J Pharm Edu.

[14] Sullivan KM, Spooner LM (2008). Adverse-drug reaction reporting by pharmacy students in a teaching hospital. Am J Health Syst Pharm.

[15] Gavaza P, Brown CM, Lawson KA, Rascati KL, Steinhardt M, Wilson JP (2012). Pharmacist reporting of serious adverse drug events to Food and Drug Administration. J Am Pharm Assoc.

[16] Gavaza P, Brown CM, Lawson KA, Rascati KL, Steinhardt M, Wilson JP (2011). Texas pharmacists’ knowledge of reporting serious adverse drug events to Food and Drug Administration. J Am Pharm Assoc..

[17] Gavaza P, Brown CM, Lawson KA, Rascati KL, Steinhardt M, Wilson JP (2011). Influence of attitudes on pharmacists’ intension to report serious adverse drug events to Food and Drug Administration. Br J Clin Pharmacol.

[18] Generali J, Danish M, Rosenbaum S (1995). Knowledge of and attitudes about adverse drug reaction reporting among Rhode Island Pharmacists. Am Pharmacother.

[19] Smith MP, Webley SD (2013). Pharmacovigilance teaching in UK undergraduate pharmacy programmes. Pharmacoepidemiol Drug Saf.

[20] Bakare M, Oreagba AI, Adeniran B (2011). Drug therapy problems: pharmacists’ interventions and outcomes in Lagos state general hospitals, South West, Nigeria West African Postgraduate College of Pharmacists (Nigerian Chapter). Newsletter.

[21] Sivadasan S, Sellappan M (2015). Knowledge and attitude towards pharmacovigilance and adverse drug reporting among dental students in a private University in Malaysia. J Young Pharm..

[22] Brown AA, Ogun JJ (1998). Clinical pharmacy practice development in Nigeria. A historical account chapter 2.

[23] Faculty of pharmacy handbook. Ile-Ife: Obafemi Awolowo University; 2016.

[24] Aguwa CN, Akubue PI, Adenike FB (2009). Pharmacy curriculum review: clinical pharmacy. Pharmacy education in Nigeria.

[25] Oparah AC (2010). Essentials of pharmaceutical care.

[26] Pharmacists’ council of Nigeria. 2016. http://www.pcn.org.ng. Accessed 25 Feb 2016.

[27] National universities commission. 2016. http://www.nuc.edu.ng. Accessed 25 Feb 2016.

[28] Fadare JO, Afolabi OA, Omokhanye HK, Enwere OO, Chedi BAZ, Musa A (2011). Knowledge, attitude and practice of adverse drug reaction reporting among healthcare workers in a tertiary centre in Northern Nigeria. Trop J Pharm. Res.

[29] Chua GN, Hassali MA, Shefel RA, Awaisu A (2010). A survey exploring knowledge and perception of general practitioners towards the use of generic medicines in the northern state of Malaysia. Health Policy.

[30] Khalili H, Mohebbi N, Hendoiee N, Kashtkear AA, Dashti-Khavidaki S (2012). Improvement of knowledge, attitude and perception of health workers about ADR, a pre- and post-clinical pharmacist interventional study. BMJ Open.

[31] Lisha JJ, Arifulla M, Cheriathu JJ, Sreedhan J (2012). Reporting of ADRs an exploratory study among nurses in a teaching hospital Ajman United Arab Emirates. DARU J Pharm Sci..

[32] Rehan HS, Vasudev K, Tripathi CD (2002). Adverse drug reaction monitoring: knowledge, attitude and practices of medical students and prescribers. Natl Med J India.

[33] Kingston R, Mari K, Shashinar N (2015). Pharmacy students’ knowledge and perceptions about ADRs reporting and pharmacovigilance. Saudi Pharm J..

[34] Oshikoya KA, Awobusuyi JO (2009). Perceptions of doctors to adverse drug reaction reporting in a teaching hospital in Lagos, Nigeria. BMC Clin Pharmacol.

[35] Guidance, compliance and regulatory information. How can a product be both a cosmetic drug?. 2013. http://www.fda.gov/cosmetics/guidancecomplianceregulatoryinformation. Accessed 13 Jan 2016.

[36] Inman WH (1996). Attitudes to adverse drug–reaction reporting. Br J Clin Pharmacol.

[37] Nwokike J. Monitoring adverse drug reactions in the public health programs: the case of the Nigeria TB programme. Submitted to the US agency for international development by the TBCAP project. 2008. http://www.apps.who.int/medicinedocs/documents/s18400en.pdf. Accessed 13 Feb 2016.

[38] Ballentine A, Mckenzie N, Wysocki A, Kepner K. The role of monetary and non-monetary incentives in the work place as influenced by career stage. 2002. http://www.edis.ifas.ufl.edu. Accessed 13 Jan 2016.

[39] Moss RL, Garnett WR, Steiner KC (1980). Physicians’ attitudes towards pharmacists counseling patients on adverse drug reactions. Am J Hosp Pharm.

[40] Aziz Z, Siang TC, Badarudin NS (2007). Adverse drug reactions predictors of under-reporting in Malaysia. Pharmacoepidemol Drug Saf.

[41] Backstrom M, Mjorndal T, Dahlqvist R, Nordkvist-Olsson T (2000). Attitudes to reporting adverse drug reactions in northern Sweden. Eur J Clin Pharmacol.

