# Supplementary material for: An evaluation of the knowledge and perceptions of pharmacy students on pharmacovigilance activities in Nigeria
Source: BMC Res Notes. 2017 Jul 12;10:273. doi: 10.1186/s13104-017-2586-9 (PMC5506577; doi:10.1186/s13104-017-2586-9)
Supplement: Supplementary file 1 — Additional file 1. Questionnaire. [file 13104_2017_2586_MOESM1_ESM.docx]

Additional file 1. QUESTIONNAIRE USED FOR THE STUDY

Section A (Demographic Profile)

Background information of final year pharmacy students

1. What is your sex? Male Female

2. Age up to 20yrs 21-24yrs 25-30yrs > 30 yrs

3. Please write the name of your university in the space below

…………………………………………………………………………………………

4. How many are you in your class? ………………………………………….

5. Have you heard of the terms adverse drug reactions and pharmacovigilance?
 Yes No

6. Did you take any pharmacovigilance course? Yes No

Section B

Evaluating the knowledge of pharmacy students about PV activities in Nigeria

Please tick on the most appropriate option.

1. What do you understand by the term adverse drug reactions ADRs?

(a) A noxious and unintended response to a drug at doses normally used in man

(b) A noxious and intended response to a drug at doses normally used in man

(c) A noxious and unintended response to a drug at abnormal doses used in man

(d) A noxious and intended response to a drug at abnormal doses used in man

2. What is Pharmacovigilance?

(a) The science detecting the type and incidence of ADR after drug is marketed

(b) The science of monitoring ADR's occurring in a Hospital

(c) The process of improving the safety of the drug

(d) The detection, assessment, understanding and prevention of adverse effects

3. Which types of ADRs should be documented?

(a) Suspected ADRs for a new drug

(b) Suspected ADRs for an old drug

(c) Suspected severe ADRs for any drug

(d) All of the above

4. Which type of reporting system do we have in Nigeria?

(a) Prescription event monitoring

(b)Spontaneous reporting system

(c) Case reports

(d) Meta-Analysis

5. All ADRs should be reported

(a)Yes

(b)No

(c) Can't say

(d) May be

6. There are no guidelines for reporting ADRs in Nigeria

(a) Yes

(b) No

(c) Can't say

(d) May be

7. What types of ADRs do you know?

(a) Types A, B, C, D & E

(b) Types A, B, C, D & F

(c) Types A, B, C, D & G

(d) Types A, B, C, D & H

8. ADRs caused by herbal medicines are neither documented nor reported

(a) Yes

(b) No

(c) Can't say

(d) May be

9. All ADRs are known before a drug is marketed

(a) Yes

(b) No

(c) Can't say

(d) May be

10. Which organization should case of ADRs be reported to in Nigeria?

(a) Pharmacists Council of Nigeria (PCN)

(b) Pharmaceutical Society of Nigeria (PSN)

(c) National Agency for Food, Drug Administration and Control (NAFDAC)

(d) National Drugs Law Enforcement Agency (NDLEA)

SECTION C

Evaluating the perceptions of pharmacy students about ADRs reporting

Indicate your level of agreement with the under-listed statements on a 5-point scale by ticking the boxes
below as SD (1) = Strongly disagree, D (2) = Disagree, N (3) = Neutral, A (4) = Agree, and SA (5) =
Strongly agree

| S/N | SURVEY STATEMENTS | SD  1 | D  2 | N  3 | A  4 | SA  5 |
| --- | --- | --- | --- | --- | --- | --- |
| 1 | I believe that I have acquired enough knowledge to enable me report ADRs. |  |  |  |  |  |
| 2 | Pharmacovigilance should be taught at all levels in pharmacy schools. |  |  |  |  |  |
| 3 | Pharmacovigilance should not be taught at all levels in pharmacy schools. |  |  |  |  |  |
| 4 | I believe that late or non- reporting of ADRs could pose major health problems. |  |  |  |  |  |
| 5 | I believe that adverse reactions caused by herbal medicines should be reported |  |  |  |  |  |
| 6 | Monetary incentives to health workers may improve ADRs reporting. |  |  |  |  |  |
| 7 | Pharmacy students can perform ADR reporting during their clerkship. |  |  |  |  |  |
| 8 | I believe that government could improve ADR reporting in Nigeria through advocacy |  |  |  |  |  |
| 9 | I believe ADR reporting is an integral part of pharmaceutical care |  |  |  |  |  |
| 10 | Pharmacists should not be barred from reporting ADRs as is the case in some countries |  |  |  |  |  |
| 11 | I believe that pharmacists’ involvement in reporting of ADRs would impact positively on pharmacovigilance activities |  |  |  |  |  |
| 12 | I believe that if the identities of ADR reporters are disclosed reporting rate will increase |  |  |  |  |  |
| 13 | I believe that if the identities of ADR reporters are not disclosed reporting rate will decrease |  |  |  |  |  |
| 14 | Pharmacovigilance should be taught only at higher levels in pharmacy schools |  |  |  |  |  |
| 15 | I believe that adverse reactions caused by cosmetics should be reported |  |  |  |  |  |
